# Major primary bile salts repress *Salmonella enterica* serovar Typhimurium invasiveness partly via the efflux regulatory locus *ramRA*

**DOI:** 10.3389/fmicb.2024.1338261

**Published:** 2024-02-12

**Authors:** Etienne Giraud, Sylvie Baucheron, Isabelle Foubert, Benoît Doublet, Kunihiko Nishino, Axel Cloeckaert

**Affiliations:** ^1^INRAE, Université de Tours, UMR ISP, Nouzilly, France; ^2^Institute of Scientific and Industrial Research, Osaka University, Osaka, Japan

**Keywords:** *Salmonella*, Typhimurium, invasion, intestinal, bile, regulation, RamR, *ramA*

## Abstract

Bile represses *Salmonella enterica* serovar Typhimurium (*S*. Typhimurium) intestinal cell invasion, but it remains unclear which bile components and mechanisms are implicated. Previous studies reported that bile inhibits the RamR binding to the *ramA* promoter, resulting in *ramA* increased transcription, and that *ramA* overexpression is associated to decreased expression of type III secretion system 1 (TTSS-1) invasion genes and to impaired intestinal cell invasiveness in *S*. Typhimurium. In this study, we assessed the possible involvement of the *ramRA* multidrug efflux regulatory locus and individual bile salts in the bile-mediated repression of *S*. Typhimurium invasion, using Caco-2 intestinal epithelial cells and *S*. Typhimurium strain ATCC 14028s. Our results indicate that (i) major primary bile salts, chenodeoxycholate and its conjugated-derivative salts, cholate, and deoxycholate, activate *ramA* transcription in a RamR-dependent manner, and (ii) it results in repression of *hilA*, encoding the master activator of TTSS-1 genes, and as a consequence in the repression of cellular invasiveness. On the other hand, crude ox bile extract and cholate were also shown to repress the transcription of *hilA* independently of RamR, and to inhibit cell invasion independently of *ramRA*. Altogether, these data suggest that bile-mediated repression of *S*. Typhimurium invasion occurs through pleiotropic effects involving partly *ramRA*, as well as other unknown regulatory pathways. Bile components other than the bile salts used in this study might also participate in this phenomenon.

## 1 Introduction

*Salmonella enterica* serovar Typhimurium (*S*. Typhimurium) is a Gram-negative enteric pathogen which causes generally localized and self-limiting gastroenteritis in humans, although some severe cases require antimicrobial treatment (Su et al., 2004; Velge et al., 2005; Giraud et al., 2006; Haraga et al., 2008). *S*. Typhimurium, after entering the gastrointestinal tract with contaminated food or water, has to overcome successive stressful environmental conditions, such as the acidic pH of the stomach or the presence of antibacterial compounds, like bile, in the small intestine (Rychlik and Barrow, 2005; Baumler et al., 2011). At each step of infection, *S*. Typhimurium needs to sense its environment and to coordinate its gene expression in order to survive host defenses and optimize its colonization. Bile, in addition to its antibacterial effect, is thus recognized by *S*. Typhimurium as an important environmental signal, whose sensing leads to important changes in the expression levels of numerous genes involved in pathogenesis (Prouty et al., 2004a; Begley et al., 2005; Rychlik and Barrow, 2005; Antunes et al., 2012).

The *acrAB* and *tolC* multidrug efflux pump genes, which are required for *S*. Typhimurium resistance to bile, are activated by bile itself (Prouty et al., 2004b; Nikaido et al., 2008). We previously reported that this occurs mainly through the transcriptional derepression of the *ramA* gene, whose product is a direct activator of these efflux pump genes (Baucheron et al., 2014). We showed in this study that bile inhibits the binding of the RamR repressor to the *ramA* promoter, however without specifying which particular bile components were involved. More recently, the crystal structure of RamR and its interaction with bile acids have been solved, identifying cholic and chenodeoxycholic acids as the most important to bind RamR (Yamasaki et al., 2019). The interaction between bile acids and RamR occurs through hydrogen bonds (Yamasaki et al., 2019).

Another important feature of the intricate interactions between *S*. Typhimurium and bile is the bile-mediated repression of non-phagocytic intestinal cells invasion (Prouty and Gunn, 2000). This invasion is largely determined by the type III secretion system-1 (TTSS-1), and some of its secreted effectors encoded by the *Salmonella* Pathogenicity Island-1 (SPI-1) (Haraga et al., 2008; Fabrega and Vila, 2013). The transcription of SPI-1 genes is tightly controlled via a complex regulatory network, which ensures that TTSS-1 and its secreted effectors are expressed only when environmental conditions are favorable for invasion (Laughlin et al., 2014). The complex network of interacting transcription factors regulating SPI-1 gene expression results in a bistability pattern (TTSS-1^ON^ and TTSS-1^OFF^ cells) in *Salmonella* populations (Hamed et al., 2019; Sanchez-Romero and Casadesus, 2021; for a review see Lou et al., 2019). This bimodal gene expression of SPI-1 has several phenotypic impacts such as growth impairment, switch in motility and increased antibiotic resistance in subpopulations (Arnoldini et al., 2014; Hamed et al., 2019; Sanchez-Romero and Casadesus, 2021). Moreover, bile was initially pointed out by Prouty et al. (2004a) as one of numerous environmental signals that help *S*. Typhimurium localize and temporally regulate the expression of invasion factors (Prouty and Gunn, 2000). The authors hypothesized that high bile concentrations present in the lumen of the anterior small bowel repressed invasion factors, whereas, for bacteria having reached the distal ileum and crossed the mucous layer of the epithelium, lower bile concentrations allowed the expression of SPI-1 genes to initiate cell invasion. They also showed that a functional BarA/SirA two-component system was required for bile sensing and for the repression of the transcription of SPI-1 invasion genes. However, the sensing of bile components by the BarA sensor kinase was not demonstrated. Other studies have shown that the expression of the SPI-1 *hilA* gene, which encodes the master activator of TTSS-1-related invasion genes, was strongly repressed by bile (Golubeva, 2010; Antunes et al., 2012). More recently, Eade et al. (2016) demonstrated that SPI-1 repression by bile acids is mediated by posttranslational destabilization of HilD, a transcriptional activator acting directly on TTSS-1 genes and indirectly by activating *hilA* transcription.

Interestingly, the *ramRA* locus, besides regulating efflux pump genes, was also suggested to be involved in the regulation of invasion genes of the type III secretion system 1 (TTSS-1) (Bailey et al., 2010; Giraud et al., 2013). Indeed, overexpression of *ramA*, either plasmid-driven or due to mutations in *ramR* or in the RamR DNA-binding site, led to decreased expression of TTSS-1 genes, including *hilA*, and to decreased invasion efficiency in some *S*. Typhimurium strains, depending on their genetic background (Giraud et al., 2013). Altogether, these observations suggested that the *ramRA* regulatory locus may possibly be involved in the bile-mediated repression of intestinal cell invasion.

In the present study, we investigated the roles of (i) individual bile salts, which are the most abundant components of bile (representing about 2/3rd of its organic content) and (ii) the *ramRA* locus in the bile-mediated repression of *S*. Typhimurium invasion. In particular, the major primary bile salts, chenodeoxycholate and cholate, as well as their derivatives conjugated with glycine or taurine, were assessed for their ability to activate *ramA* expression and to repress *hilA* expression, and as a consequence to inhibit the invasion of intestinal epithelial cells. We assessed also whether the expression changes observed with the major bile salts were dependent on *ramR*, and studied the role of the entire regulatory locus *ramRA* on the bile-mediated repression of intestinal cell invasion.

## 2 Materials and methods

### 2.1 Bacterial strains and culture conditions

*S*. Typhimurium wild-type (WT) strain ATCC 14028s and its Δ*ramR*, Δ*ramR/*p*ramR*, and Δ*ramRA*::*kan*/p*ramA* derivatives were used in this study. Deletion mutants were constructed using the Datsenko and Wanner inactivation gene method as previously described (Datsenko and Wanner, 2000; Abouzeed et al., 2008). Complementation plasmids carrying the *ramR* gene (p*ramR*) or the *ramA* gene (p*ramA*) were previously described (Abouzeed et al., 2008; Nikaido et al., 2008). Bacterial strains were grown at 37°C in Luria–Bertani broth (LB, pH 7.5) supplemented with 25.6 g/L bile or with 5 mM of individual bile salts where appropriate. Bile used in this work was a crude ox-bile extract which contains the main bile sodium salts of taurocholic, glycocholic, deoxycholic, and cholic acids purchased under the label “sodium choleate” (Sigma–Aldrich, Steinheim, Germany). Physiological concentrations of bile salts encountered by bacteria in the intestinal lumen are variable with high and low concentrations in the anterior small bowel and distal ileum, respectively, estimated in the millimolar range that is consistent with their critical micellar concentrations (e.g., 6–10 mM for taurocholic acid) (Martinez-Augustin and Sanchez de Medina, 2008). In a previous study, we showed that a bile concentration of 25.6 g/L allowed normal growth (i.e., similar to growth control in LB medium) of *S*. Typhimurium isolates (Baucheron et al., 2005). Individual bile salts (Sigma–Alrich, Steinheim, Germany), also allowed normal growth of the tested strains when used at 5 mM.

### 2.2 Invasion and adhesion assays

Invasions assays were performed as previously described (Rosselin et al., 2010). Caco-2 intestinal epithelial cells were grown in Dulbecco's modified Eagle medium (DMEM) supplemented with 10% inactivated fetal bovine serum, 1% non-essential amino acids, and 1% antibiotic solution (Gibco, Invitrogen), at 37°C under 5% CO_2_. Cells were seeded at a density of 2 × 10^5^ cells/well in a 24-well plate (Falcon) and grown until confluence in the same medium. Antibiotic was removed 24 h before the invasion assays. Bacteria grown to an OD_600_ of 0.6 in LB broth were inoculated on Caco-2 monolayers at a multiplicity of infection (MOI) of 30. After a 30 min incubation, the bacteria-containing medium was removed from the wells, and the cells monolayers were washed with phosphate buffered saline (PBS). For adhesion assays, cells were then lysed for 30 min with sterile ultrapure water and serial dilutions of lysates were plated on LB agar. For invasion assays, cells were further incubated for 1.5 h with DMEM supplemented with gentamicin at 100 μg/mL. After washing with PBS, cells were lysed with sterile ultrapure water and serial dilutions of lysates were plated on LB agar. The percentage of penetrating bacteria was calculated as the ratio of the counted colony forming units (cfu) to the bacterial inoculum. All assays were repeated at least twice, with three replicates for each tested condition. Data presented correspond to mean values of at least six replicates for invasion and adhesion assays.

### 2.3 Gene expression analysis by qRT-PCR

Bacteria were grown in 20 ml liquid cultures (standard LB, 1% NaCl), in 125 mL Erlenmeyer flasks, under shaking at 180 RPM, for about 150 min, until they reached an OD_600_ value of 0.6. Culture samples were pelleted by centrifugation, stabilized with RNAprotect Bacteria Reagent (Qiagen) and stored at −80°C until use. Total RNA was extracted using the RNeasy Mini kit (Qiagen) following the manufacturer's instructions. Residual genomic DNA was removed using the Turbo DNA-free kit (Ambion). Total RNA (1.5 μg) was reverse-transcribed using random hexamers and the Superscript III First Strand Synthesis System (Applied Biosystems). The expression level of each gene was calculated from three independent cDNA samples. For each cDNA sample and each gene, qRT-PCR runs were performed in duplicated wells. Primers and cycling conditions used for qRT-PCR were previously described (Giraud et al., 2013). The relative quantities of transcripts were normalized against the geometric mean of three reference genes (*gmk, gyrB, rrs*). Statistical significance was assessed at a *P*-value of <0.05 using a two-tailed Student's *t*-test.

## 3 Results and discussion

### 3.1 Differential effects of individual bile salts on the expression of *S*. Typhimurium ATCC 14028s invasion regulatory genes and on invasion of intestinal epithelial cells

**Figure 1:**
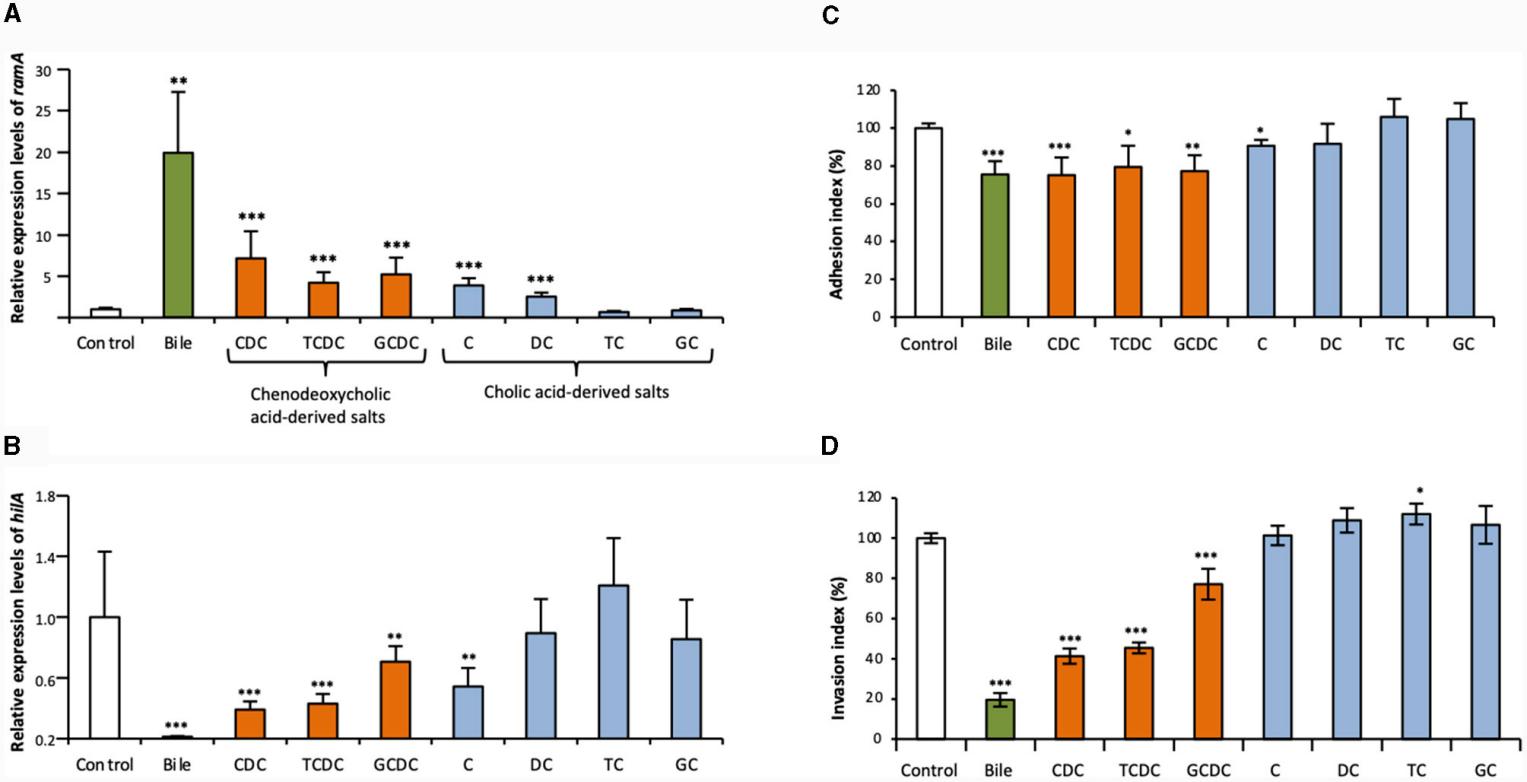
Effects of crude bile extract at 25.6 g/L or of individual bile salts at 5 mM on *ramA* and *hilA* expression and on adhesion to/invasion of Caco-2 intestinal epithelial cells. Transcript levels of *ramA* **(A)** and *hilA* **(B)** were determined using qRT-PCR for the WT *S*. Typhimurium strain 14028s. Values were normalized by those obtained for control (LB alone). Bars represent the standard deviation from three independent replicates. **(C)** Adhesion to and **(D)** invasion of Caco-2 cells by the WT strain 14028s. Results are expressed relative to values obtained for control (LB alone), arbitrarily set at 100%. Bars indicate the percentage of attached/internalized bacteria ± standard error of the mean. In all panels, asterisks indicate significant differences (*P < 0.05, **P < 0.01, ***P < 0.001). □ Controls (LB alone), 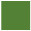 crude bile extract, 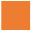 chenodeoxycholic acid-derived salts (CDC, chenodeoxycholate; TCDC, taurochenodeoxycholate; GCDC, glycochenodeoxycholate) and 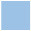 cholic acid-derived salts (C, cholate; DC, deoxycholate; TC, taurocholate; GC, glycocholate).

The qRT-PCR assays of this study confirmed that crude ox bile extract at 25.6 g/L increased *ramA* transcript levels about 20-fold, as previously described (Figure 1A) (Baucheron et al., 2014). To address the effects of individual bile salts on *ramA* expression, primary bile salts and their derivatives (dehydroxylated and glycine- or taurine-conjugated) were tested at the concentration of 5 mM. Chenodeoxycholate showed the most important effect, by increasing *ramA* transcript level ~7- vs. 4-5-fold for its taurine- and glycine- conjugated derivatives in the *S*. Typhimurium strain ATCC 14028s (Figure 1A). Cholate also increased *ramA* mRNA levels by a 4-fold factor, i.e., notably more than its dehydroxylated derivative, deoxycholate (2.5-fold). In contrast, no *ramA*-inducing activity could be detected for both cholate conjugates, taurocholate and glycocholate (Figure 1A).

In contrast to their effects on *ramA* expression, bile and individual bile salts globally showed a repressive effect on *hilA* expression in *S*. Typhimurium ATCC 14028s (Figure 1B). Furthermore, this repressive effect on *hilA* expression appeared correlated to the *ramA*-inducing effect. Indeed, crude bile extract, which showed the strongest *ramA*-inducing effect, decreased *hilA* transcripts to very low levels (about 1.5% that of the control) (Figure 1B). By contrast, bile salts with low (deoxycholate) or no (taurocholate, glycocholate) *ramA*-inducing activity, showed no significant repressive effects on *hilA* expression. Other bile salts, which induced *ramA* between 4- and 7-fold (see above), decreased *hilA* transcript levels 3–4-fold (chenodeoxycholate and taurochenodeoxycholate), 2.3-fold (cholate), and 1.6-fold (glycochenodeoxycholate) (Figure 1B). The repressive effects exerted on *hilA* by crude bile extract and by chenodeoxycholate and its conjugates were also observed for *invA*, which encodes another SPI-1 positive regulatory protein (Supplementary Figure S1). However, no significant effects of cholate and its derived salts were observed on *invA* expression (Supplementary Figure S1).

Further to above data, we hypothesized that the repression exerted by some bile salts on *hilA* expression could result into decreased cell invasion efficiency of *S*. Typhimurium ATCC 14028s. Therefore, gentamicin protection assays were performed to address the effects of bile and individual bile salts on adhesion to and invasion of Caco-2 intestinal epithelial cells (Figures 1C, D). Crude bile extract, chenodeoxycholate and its two conjugates, taurochenodeoxycholate and glycochenodeoxycholate, decreased adhesion to the Caco-2 cells by about 20% (Figure 1C). Crude bile extract decreased *S*. Typhimurium ATCC 14028s invasion about 5-fold, and chenodeoxycholate and taurochenodeoxycholate (i.e., the bile salts which most efficiently repressed *hilA* expression), decreased its invasion by about 2.5-fold (Figure 1D). These decreased invasions may be explained partly by the decreased adhesion of the strain mentioned above. It is also possible that the 20% decrease of invasion observed with glycochenodeoxycholate was mostly due to defective adhesion of the strain. Bile salts that had no significant effect on *hilA* expression did not repress invasion of the *S*. Typhimurium strain ATCC 14028s.

These data suggest that some individual bile salts actively participate to the repression of *S*. Typhimurium cell invasion by bile. They also reveal that the specific structure of bile salts may determine their activity as environmental signals to regulate gene expression and cell invasion of *S*. Typhimurium. Nevertheless, since only one *S*. Typhimurium strain was investigated, these data must be taken with caution, to avoid any overinterpretation regarding *S*. Typhimurium as a pathogenic serovar, or more generally *Salmonella* as a pathogen, since as previously published distinct genetic lineages of serovar Typhimurium may behave differently regarding cell invasion and its regulation (Giraud et al., 2013).

In our experimental conditions, chenodeoxycholate seemed to be the most active bile salt and its conjugation to notably glycine, appeared to decrease its activity. Although they do not establish any causality relationship between *ramA* activation and *hilA* repression, our results indicate that *ramA* overexpression is associated to decreased TTSS-1 genes expression and to decreased invasion of the *S*. Typhimurium strain studied. These different correlations led us to examine further the actual involvement of the *ramRA* regulatory locus in the bile-mediated repression of *S*. Typhimurium ATCC 14028s cell invasion.

### 3.2 Bile salts effects on *ramA* and *hilA* expression are dependent on *ramR* in *S*. Typhimurium ATCC 14028s

A previous study suggested that, whereas *ramA* is activated by bile mainly depending on *ramR*, another undetermined *ramR*-independent pathway also contributes to the up-regulation of *ramA* by bile in *S*. Typhimurium (Baucheron et al., 2014). Here, we assessed whether the major individual bile salts (chenodeoxycholate, cholate and deoxycholate) could induce *ramA* expression in *S*. Typhimurium ATCC 14028s by different, *ramR*-dependent and/or *ramR*-independent pathways.

**Figure 2:**
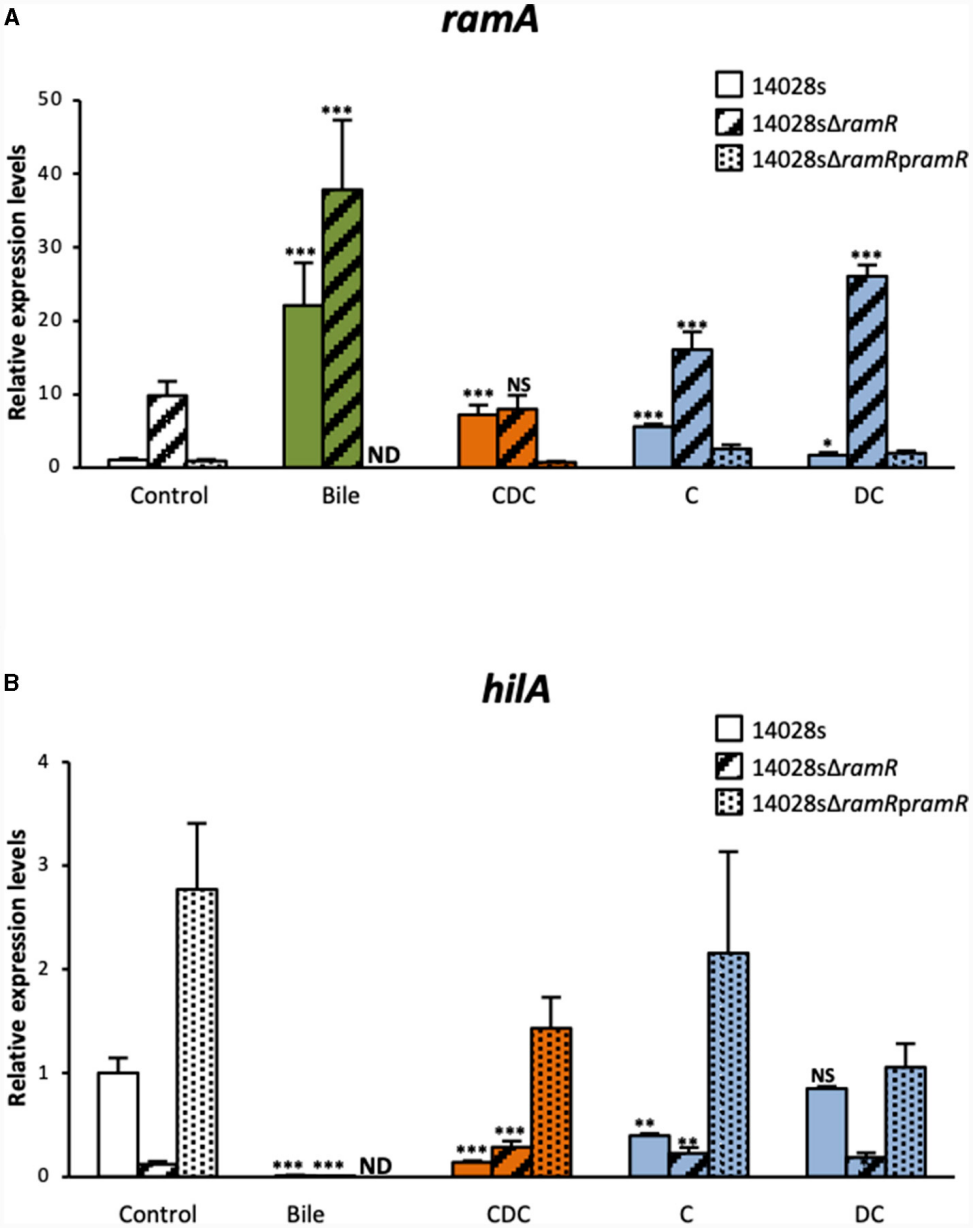
qRT-PCR analysis of the dependence on *ramR* of bile and individual bile salts effects on *ramA* and *hilA* expression. Transcript levels of *ramA* **(A)** and *hilA* **(B)** were determined for the WT *S*. Typhimurium strain 14028s strain and for its *ramR* deletion mutant, complemented or not with a p*ramR* plasmid, after growth in the presence of crude bile extract at 25.6 g/L or of individual bile salts at 5 mM. Bars represent the standard deviation from three independent replicates. □ Controls (LB alone), 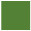 crude bile extract, 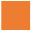 chenodeoxycholate (CDC), 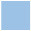 cholate (C), and 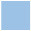 deoxycholate (DC). Asterisks indicate significant differences (NS, non-significant; *P < 0.05, **P < 0.01, ***P < 0.001). ND, not determined.

The increase of *ramA* expression in the WT *S*. Typhimurium ATCC 14028s strain, in the presence of bile or of the three tested salts, were similar to those reported above (Figure 2A). As expected, in control cultures (LB alone), the Δ*ramR* mutant expressed *ramA* transcript levels about 10-fold higher than those of the WT strain, and complementation with a functional *ramR* gene (using p*ramR*) restored WT *ramA* expression levels. Chenodeoxycholate increased *ramA* transcript levels by 7.2-fold in the WT strain, probably in a fully *ramR*-dependent manner, since no increase was observed in the Δ*ramR* mutant. Cholate increased *ramA* transcript level 5.6-fold in the WT strain. However, in contrast to chenodeoxycholate, cholate also increased *ramA* expression about 1.6-fold in the Δ*ramR* mutant compared to LB medium alone, indicating that it might also use a minor *ramR*-independent pathway to activate *ramA* expression. Lastly, the slight effects of deoxycholate on *ramA* transcript level appeared similar in the WT and in the Δ*ramR* background (1.7- and 2.7-fold increase, respectively), indicating that deoxycholate likely uses mainly a *ramR*-independent pathway to achieve this effect. Irrespective of the tested bile salt, complementation using the p*ramR* multicopy plasmid resulted in *ramA* transcript levels similar, or even lower, than those observed in the WT strain. These results indicate that the individual bile salts tested differ not only in the magnitude of their effects on *ramA* expression, but also in their relative use of the *ramR*-dependent and *ramR*-independent pathways to achieve this effect. Considering these results, the bile-mediated increase of *ramA* expression that we observed, here and before, can be interpreted as being, at least partly, the intricate result of the individual effects of bile salts, which would explain the implication of both *ramR*-dependent and *ramR*-independent mechanisms. It remains also possible that bile components other than the tested bile salts also participate to *ramA* up-regulation, depending or not on *ramR*.

In view of the negative correlation between *ramA* and *hilA* expression levels, we also tested to what extent *hilA* repression by chenodeoxycholate, cholate and deoxycholate depended on *ramR*. In control cultures (LB alone), *hilA* expression levels in the Δ*ramR* mutant were 8-fold lower than in the WT strain, in agreement with previously reported results (Giraud et al., 2013). In the WT strain, the *hilA* transcript levels were significantly decreased in the presence of chenodeoxycholate (6.8-fold) or cholate (2.5-fold), whereas deoxycholate had no effect. In the Δ*ramR* mutant (where *ramA* is overexpressed by 4–8-fold, Figure 2A), the tested bile salts did not further decrease the *hilA* transcript levels (Figure 2B). Possibly the *ramA*-mediated repression of *hilA* is saturated in the Δ*ramR* background, explaining why further increase of *ramA* expression does not result in further *hilA* repression.

Irrespective of the tested bile salt, complementation using a p*ramR* multicopy plasmid resulted in *hilA* transcript levels higher (for chenodeoxycholate and cholate) or similar (for deoxycholate) than those observed in the WT strain (Figure 2B). These results suggest that *hilA* downregulation by chenodeoxycholate and cholate is dependent on *ramR*, and via the upregulation of *ramA*. This observation is congruent with our previous study on the crystal structure of RamR and its interaction with bile acids (Yamasaki et al., 2019). Both cholic and chenodeoxycholic acids, but not deoxycholic acid, were indeed shown to bind to RamR, and more precisely through four hydrogen bonds with RamR, and to induce *ramA* expression.

Of note, crude bile extract decreased *hilA* expression to undetectable levels as well in the Δ*ramR* mutant as in the WT strain (Figure 2B). Although it is below the detection level in our experimental conditions, it may suggest that another *ramR*-independent mechanism, possibly induced by bile components other than bile salts, acts also in the bile-mediated repression of *hilA*.

### 3.3 Influence of RamA overexpression on the bile-mediated repression of cell invasion of *S*. Typhimurium ATCC 14028s

**Figure 3:**
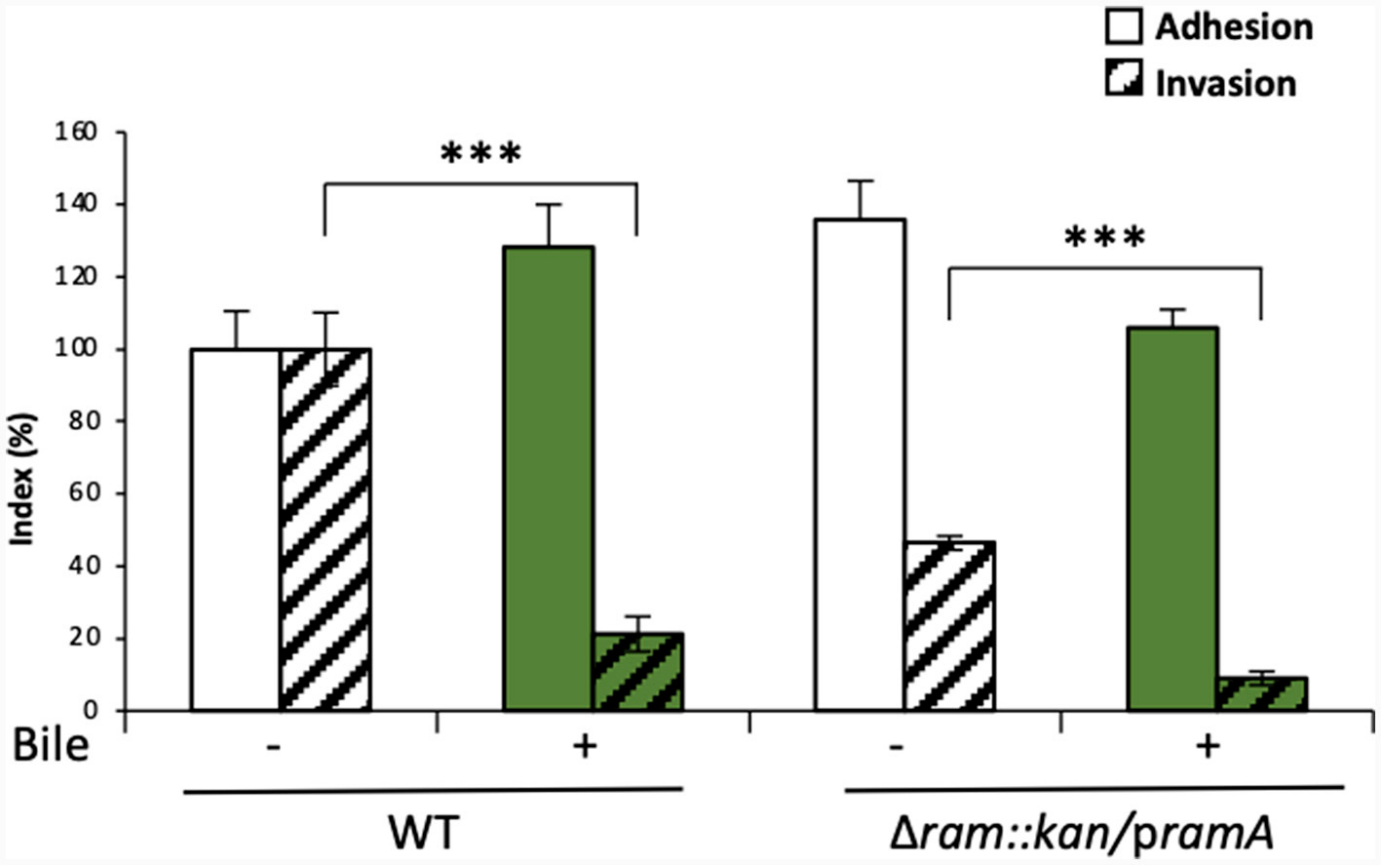
*In vitro* analysis of the dependence on the *ramRA* locus of bile effects on adhesion to/invasion of intestinal epithelial cells. Adhesion to and invasion of Caco-2 cells was analyzed after growth, in the absence (□, –) or presence (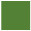, +) of crude bile extract at 25.6 g/L, of the WT *S*. Typhimurium strain 14028s and its *ramRA*::kan deletion mutant complemented with a p*ramA* plasmid. Bars indicate the percentage of attached/internalized bacteria ± standard error of the mean. Asterisks indicate significant differences (***P < 0.001).

Altogether, the results described above suggested a possible involvement of the *ramRA* locus in the bile-mediated repression of invasion, partly through RamA overexpression, resulting in repression of the master regulator *hilA* of TTSS-1 genes. To further confirm this hypothesis, the role of RamA in bile-mediated repression of invasion was tested in gentamicin protection assays, using a *S*. Typhimurium ATCC 14028s mutant overexpressing RamA (Δ*ramRA*/p*ramA*). First, invasion efficiency was confirmed to be decreased about 5-fold by bile in the WT strain (Figure 3, WT). In absence of bile (LB medium alone), ectopic overexpression of RamA in the mutant Δ*ramRA*/p*ramA* resulted in a slight increase of adhesion and in a 2-fold decrease of invasion. Using this mutant, bile further decreased invasion efficiency of an additional 5-fold (Figure 3, Δ*ramRA*/p*ramA*). These results strengthened the hypothesis that the repression of invasion occurs via different pathways dependent or not on RamA overexpression.

In sum, these results suggest that the bile-induced repression of invasion may be driven by a *ramRA*-dependent mechanism, via *ramA* overexpression, as well as through other pathways independent of the *ramRA* regulators which can alternatively have an additive effect to repress invasion. Nevertheless, the observed results must be taken with caution, because of the experimental conditions (e.g., ectopic overexpression) and the use of a single *S*. Typhimurium strain. At least, *ramRA* alone seems not entirely responsible for bile-mediated repression of *S*. Typhimurium cell invasion, and thus other genetic factors need to be further investigated.

## 4 Conclusion

**Figure 4:**
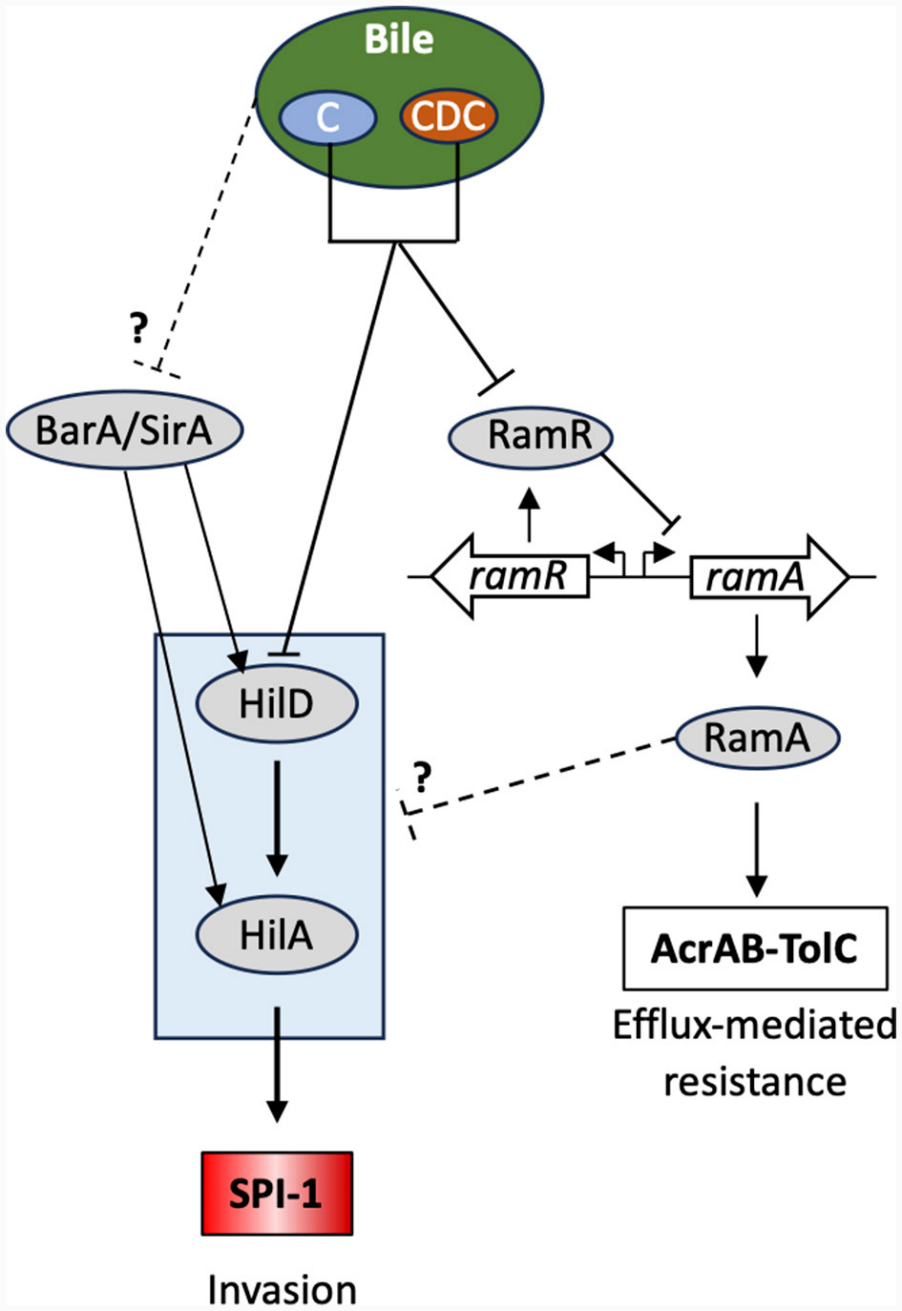
Bile-mediated regulatory network controlling SPI-1 gene expression. Only the key regulators and relevant proteins in this context are shown. Bile acids (C, cholic acid; CDC, chenodeoxycholic acid) directly bind to RamR alleviating its transcriptional repression of *ramA*. This leads to *ramA* overexpression and concomitantly repression of *hilA* and overexpression of the AcrAB-TolC efflux pump. The precise regulatory pathway between *ramA* n and SPI-1 remains unknown. See main text for further details and Lou et al. (2019) for a review.

In summary, two contrasting situations were observed, depending on whether individual bile salts or crude bile extract were used in the transcription and intestinal cellular invasion assays. On the one hand, the individual bile salts tested, mainly chenodeoxycholate or derived bile salts, were shown to activate *ramA* and to repress *hilA* in the *S*. Typhimurium strain ATCC 14028s (Figure 4). Evidence is also provided that this activation is dependent on *ramR*, and that this activation seems to vary depending on the considered bile salt, although *ramA* overexpression in any case appears to repress cellular invasion of the *S*. Typhimurium strain. In our conditions, those bile salts also repress *hilA* expression in the strain studied and its invasion of intestinal cells, likely also via the *ramRA* locus. On the other hand, crude bile extract and cholate seem also to repress *hilA* expression and intestinal cell invasion independently of *ramR* (higher repressions with the addition of bile in the Δ*ramR* genetic background), at least in our experimental conditions and for the *S*. Typhimurium strain assessed (Figure 4). This discrepancy on the dependency of the *ramRA* locus is not explained yet, but we may consider that whole bile is a complex mixture, not only by its bile salts content, but also by the presence of other molecules. Some of them may possibly counteract the activity of the one or the other individual bile salt and have pleiotropic effects at other regulatory loci than *ramRA*. In line with this, a study of Antunes et al. (2012), reported that repression by physiological bile of *phoP*, another major virulence regulator of *S*. Typhimurium, is not caused by bile salts, but rather by still unidentified small molecules present in bile. In addition, many other intestinal factors than the bile content participate in the complex regulatory network of *S*. Typhimurium intestinal cell invasion, such as intestinal fatty acid and many other small molecules found in the intestine, and interplay with the intestinal microbiota as well (Lou et al., 2019; Rogers et al., 2021; Chodhury et al., 2021a,b, 2023). Thus, further studies are needed to clarify the possible role of other bile molecules and their possible interaction in the invasion process of *S*. Typhimurium. The possible linkage(s) of the *ramRA* regulatory locus with other cell invasion regulatory loci need also to be further investigated.

## Data availability statement

The original contributions presented in the study are included in the article/Supplementary material, further inquiries can be directed to the corresponding author.

## Author contributions

EG: Conceptualization, Data curation, Investigation, Methodology, Supervision, Validation, Writing – original draft, Writing – review & editing. SB: Conceptualization, Data curation, Investigation, Methodology, Validation, Writing – review & editing. IF: Investigation, Methodology, Validation, Writing – review & editing. BD: Data curation, Supervision, Validation, Writing – review & editing. KN: Conceptualization, Data curation, Supervision, Validation, Writing – review & editing. AC: Conceptualization, Supervision, Validation, Writing – review & editing.

## Supplementary material

Supplementary Figure S1 Effects of bile and individual bile salts on *invA* expression. Transcript levels of *invA* were determined using qRT-PCR, for the WT *S*. Typhimurium 14028s strain grown in the presence of bile at 25.6 g/L or of individual bile salts at 5 mM. Values were normalized by those obtained for control samples (LB alone). Bars represent the standard deviation from three independent replicates.
