# Supplementary figures and images for: Major primary bile salts repress Salmonella enterica serovar Typhimurium invasiveness partly via the efflux regulatory locus ramRA
Source: Front Microbiol. 2024 Feb 12;15:1338261. doi: 10.3389/fmicb.2024.1338261 (PMC10895713; doi:10.3389/fmicb.2024.1338261)

Supplementary Figure S1

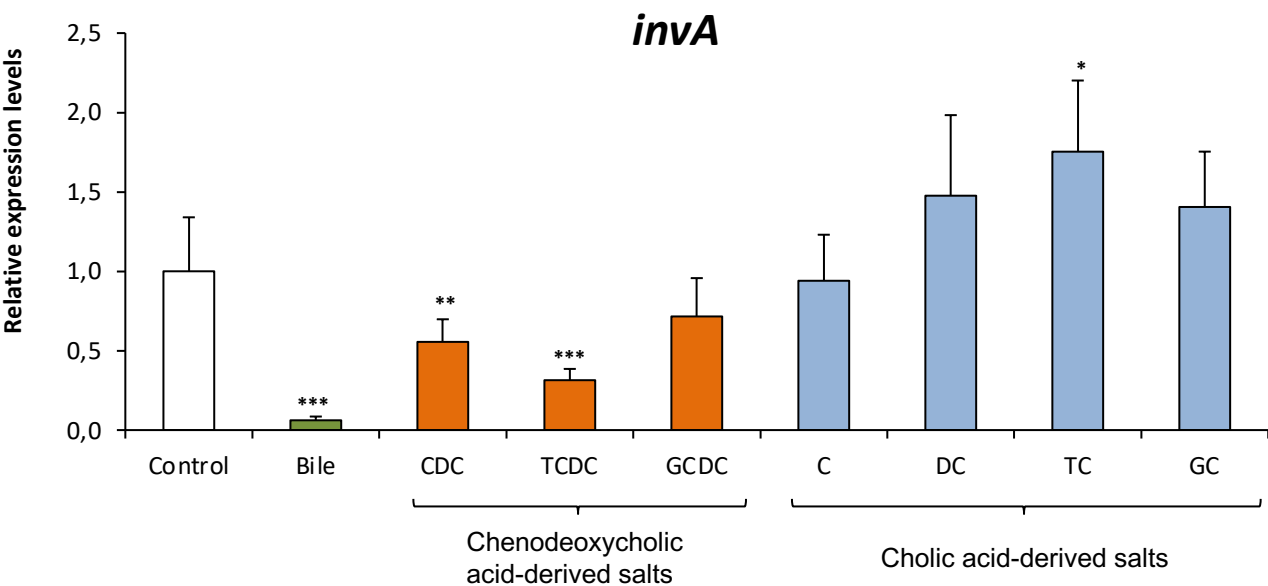

Supplement: Supplementary Figure S1 — Effects of bile and individual bile salts on invA expression. Transcript levels of invA were determined using qRT-PCR, for the WT S. Typhimurium 14028s strain grown in the presence of bile at 25.6 g/L or of individual bile salts at 5 mM. Values were normalized by those obtained for control samples (LB alone). Bars represent the standard deviation from three independent replicates. [file Data_Sheet_1.PDF]
